# Review and gap analysis: molecular pathways leading to fetal alcohol spectrum disorders

**DOI:** 10.1038/s41380-018-0095-4

**Published:** 2018-06-11

**Authors:** Friederike Ehrhart, Sylvia Roozen, Jef Verbeek, Ger Koek, Gerjo Kok, Henk van Kranen, Chris T. Evelo, Leopold M. G. Curfs

**Affiliations:** 1 Governor Kremers Centre, Maastricht University Medical Centre+, Maastricht, The Netherlands; 2 Department of Bioinformatics, NUTRIM School of Nutrition and Translational Research in Metabolism, Maastricht University, Maastricht, The Netherlands; 3 Department of Work and Social Psychology, Maastricht University, Maastricht, The Netherlands; 4 Department of Internal Medicine, Division of gastroenterology and hepatology, Maastricht University Medical Centre+, Maastricht, The Netherlands; 5 Institute for Public Health Genomics, Maastricht University, Maastricht, The Netherlands; 6 Department of Genetics, Maastricht University Medical Centre+, Maastricht, The Netherlands

**Keywords:** Molecular biology, Psychiatric disorders

## Abstract

Alcohol exposure during pregnancy affects the development of the fetus in various ways and may lead to Fetal Alcohol Spectrum Disorders (FASD). FASD is one of the leading preventable forms of neurodevelopmental disorders. In the light of prevention and early intervention, knowledge on how ethanol exposure induces fetal damage is urgently needed. Besides direct ethanol and acetaldehyde toxicity, alcohol increases oxidative stress, and subsequent general effects (e.g., epigenetic imprinting, gene expression, and metabolite levels). The current review provides an overview of the existing knowledge about specific downstream pathways for FASD that affects e.g., the SHH pathway, cholesterol homeostasis, neurotransmitter signaling, and effects on the cytoskeleton. Available human data vary greatly, while animal studies with controlled ethanol exposition are only to a certain limit transferable to humans. The main deficits in knowledge about FASD are the lack of pathophysiological understanding and dose–response relationships, together with the lack of reliable biomarkers for either FASD detection or estimation of susceptibility. In addition to single outcome experiments, omics data should be generated to overcome this problem. Therefore, for future studies we recommend holistic data driven analysis, which allows integrative analyses over multiple levels of genetic variation, transcriptomics and metabolomics data to investigate the whole image of FASD development and to provide insight in potential drug targets for intervention.

## Background

A substantial scientific knowledge about the harmful effects of alcohol consumption during pregnancy on the developing fetus exists. Fetal Alcohol Spectrum Disorders (FASD) is an umbrella term used to describe the range of birth defects caused by prenatal exposure to alcohol (which is ethyl alcohol (EtOH)). EtOH may cause mild to severe damage to the development of an unborn baby [1–6] leading to lifelong physical, behavioral, and cognitive disabilities. Depending on the nature and severity of the damage, the following diagnoses under the FASD umbrella term can be given: fetal alcohol syndrome (FAS), partial fetal alcohol syndrome (pFAS), alcohol-related neurodevelopmental deficiencies (ARND), alcohol-related birth defects (ARBD), or neurobehavioral disorder associated with prenatal alcohol exposure (ND-PAE) [1, 7–11]. Prenatal EtOH exposure can result in serious health problems affecting communities worldwide. FASD prevalence estimates range from 0 to 176.77 per 1000 livebirths [12]. FASD is in fact fully preventable, as EtOH consumption during pregnancy can be avoided. FASD is therefore one of the most important preventable forms of non-genetic birth defects associated with intellectual disability [13–16].

EtOH is metabolized in two major ways [17]: by ADH (alcohol dehydrogenase) and CYP2E1 (cytochrome P450 2E1) pathway and to a lesser degree by catalase (CAT). ADH is a cellular enzyme which is responsible for about 90% of EtOH clearance. CYP2E1 is located in liver and brain and responsible for about 10%, unless the EtOH concentration rises. ADH (Michaelis–Menten constant (K_M_) = 4.5 mg/dl) is saturated much earlier than CYP2E1 (K_M_ = 74 mg/dl). In human fetus CYP2E1 is active from week 16, ADH only from week 26; both have much lower enzyme levels and activity than adults [18]. Due to accumulation and lower clearance of EtOH in the fetus (or embryo), concentrations are higher and longer lasting in the fetal environment.

EtOH and its catabolite acetaldehyde are toxic themselves, but according to the current knowledge oxidative stress is the major damage pathway. ADH and CYP2E1 (and to a much lower degree CAT) catalyze the same reaction from EtOH to acetaldehyde, but CYP2E1 produces reactive oxygen species (ROS) as side products. In an uncontrolled manner ROS oxidize lipids, proteins, and other metabolites, and cause DNA damage (see also DNA damage response pathway [19]). Increased DNA damage triggers apoptosis pathways leading to neurodegeneration. Serotonergic neurons seem to be especially susceptible to EtOH-induced apoptosis [17]. These apoptosis events have been correlated in the decrease of brain volume and abnormalities of cortical structures that lead to alterations in cognition and behavior [20].

The cellular pathways to clear ROS (which also occur during mitochondrial respiration and several other normal parts of metabolism) involves a battery of enzymatic and non-enzymatic pathways including SOD, CAT, GPx, (reduced) glutathione, and several antioxidant metabolites (e.g., tocopherol, melatonin). Application of antioxidants have been shown to rescue some EtOH toxicity-induced phenotypes in vitro but, to date, in vivo application have not been as successful, possibly due to the insufficient bioavailability at the point of need [18].

In particular, the developing brain is susceptible to damage due to elevated ROS levels. First, it has the highest oxygen metabolic rate of all body tissues. Second, it is rich in unsaturated fatty acids and auto-oxidazible neurotransmitters that are substrates for ROS [21]. Third, the reaction with ROS generates superoxide, quinones, and semiquinones, which are again highly reactive radicals. Fourth, the levels of antioxidant enzymes are lower in brain than in other tissues (SOD, CAT, GPx). Finally, fetal cells are more in danger than adult ones because of general lower amount of EtOH degrading enzymes. Several disorders are caused or triggered by oxidative stress, nevertheless, FASD phenotype shows some distinctive features which occur in that combination only in FASD. Therefore, the downstream effects of EtOH, oxidative stress towards permanent, and long-term influence in the developing brain needs to be elucidated to understand the etiology thoroughly.

The aim of this paper is to provide a current status and a gap analysis of FASD knowledge with a focus on molecular pathways. We elucidate the downstream effects of EtOH exposure from basic alcohol metabolism towards the clinical phenotype of FASD via gene expression and epigenetic imprinting changes and give a thorough analysis of which data and information is currently available and what is currently missing. Special emphasis is laid on data driven research using high throughput methods.

## Elucidating the downstream effects of EtOH exposure

**Fig. 1:**
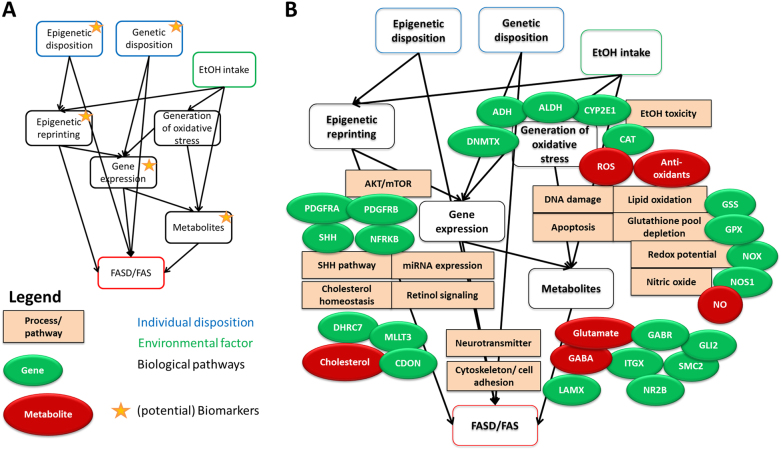
**a** Factors influencing FASD outcomes. Individual genetic disposition and environmental factors trigger the biomolecular mechanisms that lead to disease development. Biomarkers are possible in any of the molecular data domains. **b** Known biological processes/molecular pathways, major genes, and metabolites in EtOH downstream effects leading to FASD

There are several downstream effects of EtOH in the developing fetus depending on individual exposure and disposition (Fig. 1a). Firstly, the reaction on alcohol metabolism results in changed gene expression, and thereafter long-term changes in epigenetic imprinting which even can last over generations [22]. Secondly, susceptibility of individuals to increased EtOH intake and possibly FASD development is influenced by genetic and epigenetic disposition. Certain active alleles of ADH are less frequently found in mothers of FASD children, and there are also variants of ALDH and CYP2E1 suspected to have an impact on disease development [23].

### Small molecule metabolism

The main molecules generated in EtOH metabolism are EtOH and acetaldehyde. Side products of their metabolism are short living oxygen radicals or longer living molecules that originate from different reactions with other metabolites. Such metabolites from EtOH metabolism are frequently used as biomarkers as they are easy to measure in body fluids. It would be desirable to detect FASD endangered children as early as possible to be able to interfere with both psychological support and possibly pharmacological treatment. Generally, there are two major classes of biomarkers in EtOH exposure research, direct alcohol biomarkers (alcohol metabolites) and indirect alcohol biomarkers based on alcohol induced cell or tissue damage.

Direct alcohol biomarkers:

- Fatty acid ethyl esters (FAEEs). There is a trend shown towards a correlation between EtOH intake and FAEE deposition in a newborns meconium with very high maternal consumption rates, but there is no correlation in low amounts. Additionally, the variation across studies is very high [24, 25].
- Ethyl glucuronide (EtG) is a side product of EtOH metabolism and formed in the liver. It can be detected in urine for only up to two to three days, and due to its high sensitivity, urinary EtG may be positive after accidental consumption of foods containing alcohol. Scalp hair EtG is a highly sensitive and specific biomarker of chronic excessive alcohol use [26].
- Ethyl sulfate (EtS), another liver side product of EtOH, has a detection period of even less (30 h).
- Phosphatidylethanol is generated from a phospholipid in presence of EtOH and can be detected in blood up to three weeks. The available test is highly sensitive and also moderate drinking behavior (less than 40 g/day) can be detected.
- Urinary dolichol [27], but questioned by experiments of Stetter et al. [28].
- Serum *γ*-glutamyl transferase activity [29].
- Certain isoforms of alcohol dehydrogenase and *α*1-antitrypsin [30].

Indirect alcohol biomarkers [31]:

- g-Glutamyltransferase (GGT): a liver enzyme which is elevated in blood after chronic exposure to alcohol.
- Mean corpuscular volume (MCV): mean red blood cell volume, increases with chronic alcohol consumption.
- Carbohydrate-deficient transferrin (CDT): measured as percentage CDT of total transferrin, elevated levels after moderate to severe alcohol consumption, but limited sensitivity.

Goldberg et al. reviewed the metabolomics studies in FASD and recommends putting more emphasis on omics data profiles, e.g., biological pathways, instead of single metabolite markers. Specific patterns were found for both human (placenta samples) and animals in fatty acid/sphingolipid and amino acid metabolism (namely Tyrosine and Tryptophan) [32]. Bahkireva et al. summarized the different biomarkers and their temporal window of detectability [31].

### Gene expression

Gene expression changes after EtOH exposure can be differentiated in short-term and long-term changes.

On short-term often oxidative stress [33], energy metabolism, and apoptosis genes are affected, which lead to decreased cell proliferation and cell survival on cellular level (due to increased apoptosis) [34]. EtOH causes dysregulation of mitochondrial bioenergetics in neuronal cells leading to inhibition of mitochondrial proliferation and differentiation, reduction of mitochondrial volume, decrease of the activity of the mitochondrial respiratory chain complexes, and ATP synthase. This leads to a general reduction of ATP and depletion of mitochondrial GSH (reduced glutathione). Other observations are negative effects on homeostasis in general, e.g., via the FOXO pathway [35].

Long-term effects include changes in the levels of growth factors, cytoskeleton, cell adhesion molecules, and in the neurotransmitter system [18]. These are mainly pathways that coordinate growth, structure, and function of the central nervous system [36], but other organs (heart, kidney, and immune system) are also involved [37]. Especially neuronal crest formation is affected probably due to destabilization of β-catenin [38]. Furthermore, EtOH itself and oxidative stress-induced downstream pathways cause changes in DNA methylation (see also cytosine methylation pathway [39]), leading to changes in epigenetic imprinting. This causes long-term changes in gene expression leading to altered brain structure and function. Initial studies indicate that these methylation changes are also found in the germ cells (and offspring) of EtOH-exposed males [22].

There are several omics data driven approaches to investigate the downstream effects of EtOH-induced damage, which lead to the distinct phenotype of FASD. These investigations can be done by (1) analysis of omics data to reveal the differently expressed genes, differentially affected molecular pathways, or biological processes (e.g., by performing pathway analysis or gene ontology analysis). By gene expression analysis of rat placenta Rosenberg et al. [40] identified 22 genes whose expression at term is significantly altered in alcohol-consuming dams. Another proteomics study showed an increase in α-fetoprotein expression [41]. The function of α-fetoprotein is not yet fully understood, but it had been observed as a marker of several embryonic stress situations. A highly interesting transcriptomics meta-study revealed a massive downregulation of 104 genes [42]. Several of those are involved in RNA management (e.g., splicing, start of translation) and chromatin organization.

(2) An alternative approach is the investigation of the molecular causes of certain phenotypes which occurs in other disorders and investigation of their potential overlap with genes and pathways that are affected in FASD, too. Starting with a known phenotype and investigation of the known underlying genetic causes, the Hedgehog pathway was discovered to be disrupted in prenatal EtOH exposure. Especially the two major genes, SHH and GLI2, are responsible for some more (holoprosencephaly) or less severe phenotypes (cleft palate) of which prevalence is increased within the spectrum of FASD (or EtOH-exposed fetus, respectively) [43]. Certain variants of SHH and GLI2 were found in animal models susceptible to EtOH-induced skeletal damage, indicating that the degree of damage is dependent on the genetic background as well [44]. Another approach performed by Lombard et al. is based on the integrational approach of combining literature and prior knowledge databases to identify potential affected genes and found TGF-β, MAPK, and Hedgehog signaling pathways to be potential candidates for disease causing pathways [45].

### Long-term epigenetic effects

#### DNA methylation

Generally, global hypomethylation of DNA was observed in ethanol exposed rodents due to a direct effect of EtOH on the one-carbon pathway [46]. EtOH inhibits directly MTR (5-methyltetrahydrofolate-homocysteine methyltransferase), SLC19A1 (solute carrier/folat transferase), and MAT1A (methionine adenosyltransferase 1A). A study on Agouti mice found that the offspring from mothers exposed to alcohol shifted towards yellow coats indicating hypomethylation of Agouti promoter [47]. Systematic decrease of methylation levels of promoter regions were identified by MEDIP-chip and matched with decreased gene expression levels [48]. Decreased methylation levels in this study were directly correlated with higher levels of neural tube defects.

DNA methylation patterns are generally long living but not static. Especially during developmental processes, they undergo characteristic changes and these processes are disturbed by alcohol as monitored by Chen et al. for the hippocampal areas conus ammonis and dentate gyrus [49]. Nevertheless, several EtOH-induced altered methylation patterns are inherited by the following generations as studies in mice showed by Abbott et al. [50] leading to changed gene expression and phenotype (e.g., body weight, brain weight, and anxiety behavior) in the F1–F3 generations.

#### Histone modifications

Alcohol exposure influences histone modifications in several ways. Generally, histone methylation is reduced and acetylation is increased [51]. Specific enrichment was found for H3K9ac, H3K27me2,3, and H3K9me2 and increased expression of histone deacetylases (HDACs) and histone methyltransferases (EHMT2) [52].

Addition of acetyl groups removes positive charge of Lysine so the histone becomes less positive and this weakens the binding to the negatively charged DNA. Acetylation levels are controlled by HATs (histone acetyl transferases) [53] and HDACs. Ethanol metabolism increases acetate levels and ethanol directly inhibits HDACs.

Histone methylation occurs primarily at Lys and Arg residues, whereas mono or multiple (di or tri-) methylation of a histone protein is possible. Methylation has no effect on charge, but influences DNA binding via effector proteins, e.g., H3K4me3 linked to the initiation of transcription acting like a switch. Histone methyltransferases are dependent on SAM (S-adenosylmethionine) which levels are altered by ethanol metabolism [52].

Histone modifications are less stable than DNA methylations and are not well characterized in disease. Some modifications can be inherited through cell division, whereas the exact mechanism for how histone modifications are copied after DNA strand split is unclear.

## Conclusion and gap analysis

### What do we have and what is missing

In short, the main deficits in knowledge about FASD are that there is no clear pathophysiological understanding, no cure, no dose–response curve for EtOH intake during pregnancy, and no reliable biomarker for FASD detection and assessment criteria of individual susceptibility. Currently, research on FASD includes too many variable factors that overlap and confound the results to allow drawing clear conclusions. These factors include the following:

- EtOH intake: amount and timing, drinking behavior (binge or events)
- Genetic disposition: alleles of ADH, CYP2E1 etc.
- Epigenetic disposition and modifications
- Maternal body profile: age, weight
- Nutrition and lifestyle: amount of antioxidants, fatty acids, iron, exercise
- Drugs/medication
- Comorbidities that involve oxidative stress: cardiovascular diseases, atherosclerosis, cancer, diabetes, toxicity of heavy metals, radiation injury, vitamin deficiency, and inflammation (bacterial or viral infection, autoinflammatory processes) [29].

Animal studies give valuable insight in the mechanisms but are not fully translatable to humans. In humans, nevertheless, for elucidating the pathology, investigation of potential biomarkers and exploration of treatment options, there is a lack of clinical evidence.

Figure 1b visualizes the pathways and molecules currently known to be involved in FASD development. Starting from this knowledge, there are five key areas to which more research should be devoted. Below, each area is briefly described with mention of the gaps in the knowledge and the opportunities for more research.

1. Metabolites/metabolomics data. Areas of interest are (1) metabolites of ethanol metabolism and (2) metabolites of ethanol-induced pathology. Due to their easy availability in body fluids, meconium or hair, metabolites are highly interesting to yield biomarkers not only for EtOH consumption behavior, but also for early FASD detection. As single metabolites tend to fail in detection of low/medium EtOH intake, especially long after EtOH intake, multi-metabolite (or metabolomics) profiles could provide higher sensitivity and specificity. Another open question concerning EtOH metabolism is still whether there are reactive nitrogen species involved in generation of oxidative stress. Furthermore, it is unknown whether any of these biomarkers could be used for diagnosis.
2. Gene expression data. Transcriptomic and proteomic changes are direct downstream effects of ethanol. There have been initial reports mentioning that gene expression profiles are significantly changed, and a meta-study even indicated systemic downregulation of gene expression [42]. Yet, there are several open questions: is this general downregulation of gene expression reversible? Is it influenceable by drugs? Which gene expression profiles could be used as biomarkers especially to detect low and medium EtOH intake? And what further insight into the mechanisms of EtOH-induced pathology can be obtained from gene expression profiles?
3. Epigenetic data. There is some evidence (1) that epigenetic processes are involved in disorder development and progression, as ethanol influences DNA methylation processes and (2) that epigenetic changes due to ethanol influences in parents play a role in embryonic development and might be inherited in the following generation. However, the mechanisms underlying these effects are not yet fully understood. There are drugs available which influence DNA methylation events; whether they have a potential to restore FASD imprinting remains to be elucidated. All studies of histone modifications have been done in mouse models and the mechanism for how modifications are copied into new set of histones is unclear [52].
4. Genetic/genomic data. The genetic background of FASD susceptibility is not yet fully understood but there are hints that different polymorphisms of ADH, CYP and taste receptors play a role. GWAS studies have not yet been carried out on FASD.
5. Linked data. Linked data and especially FAIR data (Findable, Accessible, Interoperable, Reusable) [54] is especially useful for fields of research where little primary data is available as in the case of rare disorders. Collection of data from different sources, combining, modeling (prediction), manipulating, extending data, and re-analyzing it, there is a lot of add-value of smaller studies [55]. There is currently no public database for FASD-related data, but there are several local (clinical) databases, which could be the starting point.

### Treatment potential

As oxidative stress is likely a major pathway of EtOH toxicity in FASD, antioxidants would be the logic treatment of choice. There have been several studies demonstrating the successful rescue of EtOH-induced phenotypes in vitro and in animal models using Vitamins C and E, folic acid, glutamine, boric acid, choline, or selenium. Unfortunately, such treatment showed no significant effects when used in humans [18]. Generally, the use of antioxidants to treat oxidative stress-related diseases is highly questionable as the most reliable reviews indicate a lack of effect when applied in human patients (e.g., gastrointestinal cancer [56], age-related cataract [57], or liver diseases [58]).

Another possibility would be to look at the downstream pathways for drug targets/interventions. EtOH is known to interfere with several biological pathways, e.g., the cholesterol-SHH pathway. Experiments in zebrafish demonstrated that supplementation with cholesterol can rescue the phenotype [59]. Similar positive results were achieved in rats using metformin to interfere with the DNA methyltransferase 1 pathway [60]. DHM, a GABA receptor antagonist has been shown to neutralize EtOH effects on GABA receptor pathways and has some protective effects in rats [18]. Neuroprotective peptides and neurotrophic growth factors were also investigated with some success in vitro [18]. Whether these approaches are successful in situ remains to be elucidated.

Chokroborty-Hoque et al. [61] stated that brain development goes on after birth (and ends at adolescence), so there is time and possibility for improvement. These researchers proposed that antipsychotic drugs (which includes antidepressiva or stimulants) could be used to treat intellectual disabilities including psychosis via changes in DNA methylation. They also encourage psychological interventions (“post-natal enrichment” therapy) to treat FASD.

FASD is a widespread problem in many countries across continents. The costs to the society and to the health system are significant. Given the serious consequences of prenatal alcohol exposure with its serious effects on the affected individual, the family, and the society, priorities should be given to improve our current pathophysiological understanding of FASD and to develop strategies for preventive management and treatment to reduce or eliminate harmful effects of alcohol exposure.
